# A Rare Case of Squamous Cell Carcinoma of the Cervix With Incisional Site Recurrence

**DOI:** 10.7759/cureus.21447

**Published:** 2022-01-20

**Authors:** Anjuman Sultana

**Affiliations:** 1 Gynecologic Oncology, Directorate General of Health Services, Dhaka, BGD

**Keywords:** recurrence, scar, incision, cervix, carcinoma

## Abstract

Incisional site metastasis in abdominal malignancy following laparotomy is uncommon and extremely rare in cervical cancer. The prognosis is poor in these patients, as they also present with distant organ metastasis. We report a case of squamous cell carcinoma of the cervix in a 40-year-old woman primarily treated with radical hysterectomy and concurrent chemoradiotherapy. Later on, she presented with incisional scar site recurrence and underwent surgical excision of the metastatic site several times along with chemotherapy. She has been in stable condition for nearly 10 years since her first recurrence. We highlighted the management strategies of this patient that contributed to her longevity.

## Introduction

Cervical cancer is the fourth most common cancer in women around the world and the fourth leading cause of cancer death. Its incidence is higher in developing countries, which indicates lower coverage of cervical cancer screening programs [1]. More than 270,000 women die of cervical cancer every year, more than 85% of whom die in low- and middle-income countries [1,2]. According to the 2020 National Comprehensive Cancer Network Guidelines, the treatment of cervical cancer depends on the cancer stage classified using the 2018 Federation Internationale de Gynecologie et d’Obstetrique or FIGO guidelines. The treatment modalities include surgery, surgery with adjuvant chemoradiotherapy (CRT), definitive CRT (surgery not indicated), and neoadjuvant CRT with surgery [2].

Most recurrences occur within 2 years of diagnosis. The causes of death in these patients include uremia, infections, hemorrhage, peritonitis, and organ metastasis. Following primary surgery or radiotherapy in women with early stage (stages IB-IIA) cervical cancer with no lymph node involvement, the recurrence rate is around 10%-20%, whereas the relapse rate of those with nodal metastases and/or more locally advanced tumors is up to 70%. The most common recurrence sites are the parametrium, pelvic lymph nodes, and vagina. Distant metastasis is usually found in the lung, bone, and liver. The incidence of incisional scar recurrence is very low (0.1%-2%) [2,3], and Sharma et al. have documented a case report of incisional scar metastasis in carcinoma cervix patient in 1981 that was first reported by Greenlee et al. [4]. Operative wound metastasis in cervical carcinoma has been reported in abdominal incisions, laparoscopic port sites, episiotomy incisions, and drain sites [4].

Recurrent cervical cancer is challenging for both patients and oncologists due to limited therapeutic options. The overall survival is usually less than 1 year [4]. The case described here involves recurrent carcinoma in the cervix for which surgery and chemotherapy were the mainstays of treatment.

## Case presentation

A 40-year-old female patient with diabetes and hypertension presented with irregular per vaginal bleeding. She was diagnosed with invasive squamous cell carcinoma of the cervix stage 2A grade I in 2004. Wertheim hysterectomy with bilateral salpingo-oophorectomy with bilateral pelvic lymph node dissection was performed via a Maylard incision. The histopathology report showed invasive squamous cell carcinoma of the cervix grade II with a 2-cm tumor without any nodal involvement. She received adjuvant radiotherapy in the same year.

The patient was on regular follow-up. In 2006, she had her first recurrence and developed a 4 × 5 cm mass in the right iliac fossa, which was along the incision line. The fine needle aspiration cytology (FNAC) report from the mass indicated metastatic, poorly differentiated, non-keratinizing squamous cell carcinoma. Computed tomography (CT) showed no other organ involvement. Wide excision of the parietal mass and reconstruction of the abdominal wall with a Prolene mesh was performed. The histopathology report reflected the FNAC report findings. The resection margin was negative. The patient received no chemotherapy at that time, and the case was uneventful 5 years thereafter.

In November 2012, the second recurrence occurred, and the patient presented with a lower anterior abdominal mass. CT revealed a neoplastic, lobular, soft-tissue mass in the pelvis extending into the anterior abdominal wall with left external iliac lymph node enlargement. She was again admitted for laparotomy in 2013. Perioperative findings were growth infiltrating the urinary bladder and sigmoid colon along with the lower anterior abdominal wall. Wide local excision was performed with a terminal colostomy to avoid future obstruction. The histopathology report found on gross examination an abdominal mass measuring 16 × 10 × 5 cm with muscle involvement. Microscopic examination indicated poorly differentiated squamous cell carcinoma. Postoperative wound infection developed and was treated by daily wound dressing. Six cycles of chemotherapy with carboplatin and paclitaxel were prescribed with palliative intention. The patient remained symptom-free until 2019.

**Figure 1:**
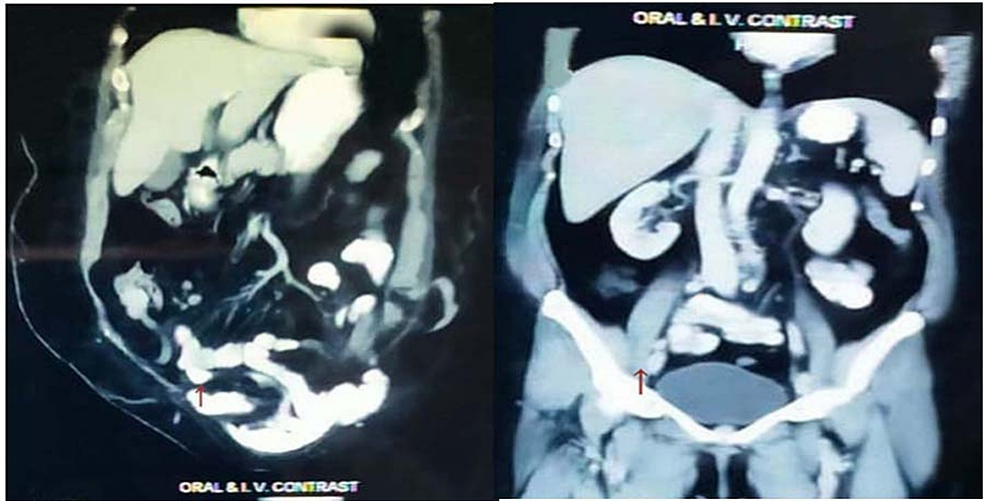
Follow-up computed tomography in sagittal and coronal view of the whole abdomen. CT showing the hyperdense area in colostomy site and vaginal stump suggestive of metastatic deposits (arrows), rest of the abdominal organs were normal.

In 2019, the patient returned, complaining of bleeding during defecation. Imaging showed metastasis in the colon, liver, and lung. The colonoscopy report found nodularity at the blind end of the colon, 25 cm from the anal verge. Biopsy of the sigmoid colon showed squamotransitional carcinoma invading into the lamina propria. P63 immunohistochemistry returned positive and indicated metastatic squamous cell carcinoma. The patient again underwent six cycles of carboplatin and paclitaxel. Post-chemo CT 2019 scan showed there was metastatic deposit in colostomy and vaginal stump area but no lymph node and other organ involvement (Figure 1).

**Figure 2:**
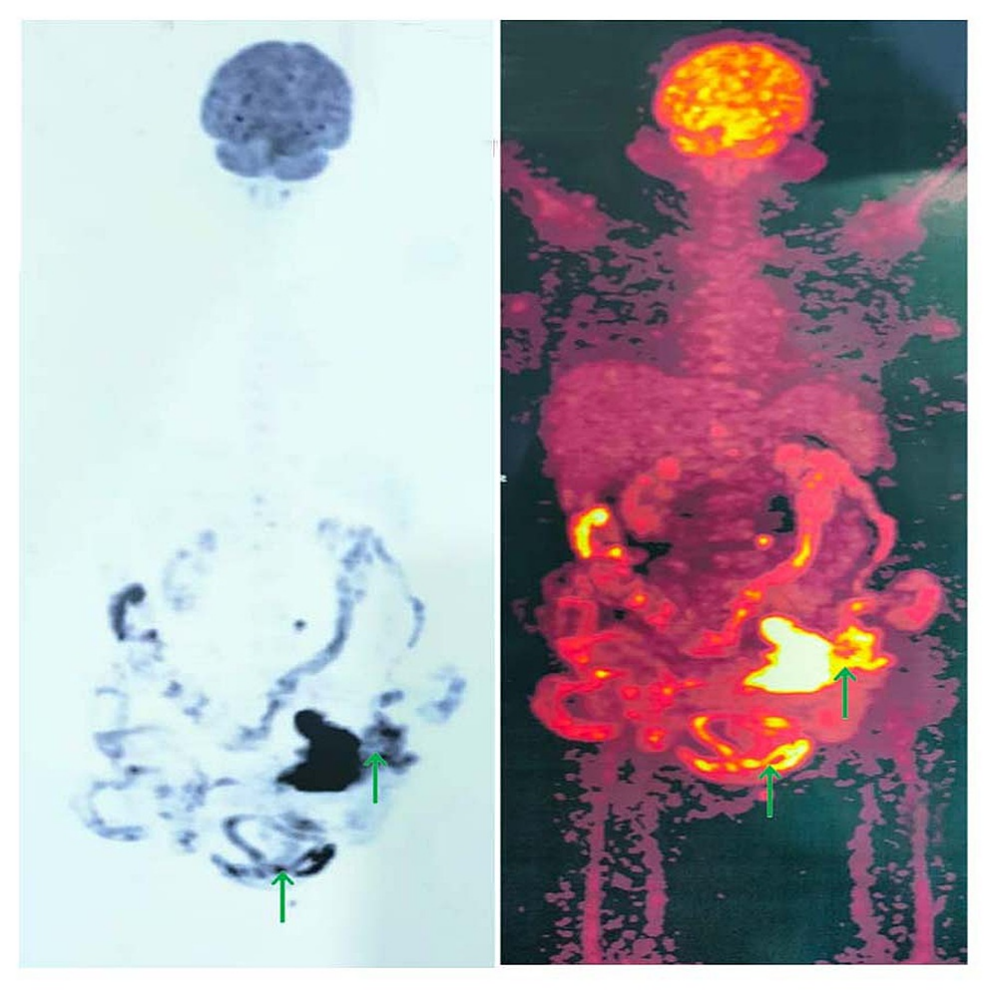
PET-CT scan showing physiological uptake of fluorodeoxyglucose (FDG) in the brain, head, neck, and thorax. FDG avid heterogeneously enhancing ill-defined lesion involving sigmoid colon, left superior-lateral aspects of urinary bladder, left lower anterolateral abdominal wall with adjacent muscles, and para-aortic and right inguinal lymph nodes suggestive of metastatic deposits. PET: positron emission tomography

MRI in early 2020 showed metastasis in the colon and urinary bladder but no liver involvement. Comparing the 2019 post-chemotherapy CT and 2020 MRI reports, it was revealed that the patient was in stable condition without further disease progression until the time of writing. PET-CT scan performed in December 2020 showed metastasis in para-aortic and right inguinal lymph nodes, urinary bladder, vaginal stump, and no metabolically active malignant disease elsewhere in the body (Figure 2). The patient had no symptoms of metastasis and the tumor board comprising oncologist and gynae oncologist decided not to do further intervention except follow-up as she had no complaints.

In brief, this patient has experienced scar site recurrence three times for which she has undergone surgery two times: the first time without chemotherapy and the second time with chemotherapy, and lastly, third time, she received chemotherapy with palliative intension.

## Discussion

Squamous cell carcinoma of the cervix comprises 70%-78% of cervical malignancies. The recurrence rates of cervical carcinoma range from 30% to 60%. Margin and node positivity, advanced stage, poorly differentiated tumor, lymphovascular space invasion, and deep stromal invasion are associated with an increased risk of recurrence. Recurrence may be symptomatic or asymptomatic. Patients commonly present with vaginal bleeding, back pain, leg edema, hematuria, weight loss, and abdominal lumps. Forty-one percent of recurrences occur within 1 year [2,5]. This case presented with an abdominal lump and recurrence in the superficial fascia at the lateral margin of the rectus abdominis muscle within 2 years after treatment. Lymphatic drainage of this location was through the inferior epigastric chain.

Because of the rarity of incisional cancer recurrence, the optimal management of this condition is still unclear. Treatment is individualized and depends on the extent of the disease and the presence of metastasis [2,5]. The patient in the present report had the first recurrence at the incisional site, which was successfully treated with wide local excision without chemotherapy. A study found that the 5-year survival rate was 62% in patients who underwent locally extended endopelvic resection with a negative margin for recurrent cervical carcinoma [6]. Cut margin negativity and absence of other organ metastasis probably contributed to the patient’s subsequent recurrence-free survival of 6 years. The patient underwent six cycles of carboplatin and paclitaxel for the second and third recurrences, with a time interval of 6 years, which implies this case was platinum sensitive. Matoda et al. defined the platinum-free interval of more than 2 years as the cutoff point between platinum sensitivity and resistance in cervical cancer [7]. Pectasides et al. evaluated the response to carboplatin and paclitaxel in recurrent cervical cancer and found that the complete response rate was 16%, the median progression-free survival was 6 months, and the median overall survival was 13 months with an acceptable toxicity profile [8]. Furthermore, patients with scar recurrence at other sites in cervical carcinoma were also treated with cisplatin with 5-fluorouracil and doxorubicin, along with radiotherapy. Chemotherapy with paclitaxel, ifosfamide, and cisplatin was well tolerated in one study [9].

This report also reviewed different case reports and found that the oncologic outcomes in cervical cancer with surgical scar metastasis are generally poor, with the length of survival ranging from 1 to 37 (mean: 8.5) months. Only one case study reported survival of more than 10 years [10]. Frequent follow-ups, at least in the first 2 years after the initial primary treatment, are highly recommended. Patient counseling, careful and regular follow-up, early detection of metastasis, and timely and appropriate intervention is emphasized for long-term survival. Identification of platinum-sensitive cervical cancer patients should be also considered in future research.

## Conclusions

Several aspects of cervical cancer are currently being actively explored with the aim of improving the treatment of cervical cancer, especially decreasing the recurrence rate. Nearly all patients with recurrence still succumb to cancer. Surgical excision remains the primary treatment for scar site recurrence as long as there is resectability in post-irradiated cervical carcinoma. Better treatment strategies are undoubtedly still needed. Once scar metastasis is noticed, management strategy should be individualized depending on the extent of metastasis, resectability, and sensitivity to platinum-based chemotherapy. Future progress in the treatment of cervical cancer and its recurrence will be obtained from continued participation in appropriate clinical trials.
